# Correction to “Association of Cannabis With Chronic Obstructive Pulmonary Disease and COVID‐19 Infection”

**DOI:** 10.1002/cdt3.70010

**Published:** 2025-05-15

S. Lehrer and P. H. Rheinstein, “Association of Cannabis With Chronic Obstructive Pulmonary Disease and COVID‐19 Infection,” *Chronic Diseases and Translational Medicine* 8 (2022): 238–241, https://doi.org/10.1002/cdt3.38.

The original article included a Conflict of Interest statement which stated: “The authors declare no conflicts of interest.” The journal and the publisher have determined that a conflict of interest on behalf of each author was not declared at publication. The journal and the publisher have had sufficient communication with the authors via email after raising questions about the Conflict of Interest in the article. The authors declared that there was no undeclared Conflict of Interest and further stated that their individual affiliations with commercial entities did not influence the research findings. Following further correspondence between the publisher and the authors, all parties have agreed to include the following Conflicts of Interest statement with the published article.

**Conflicts of Interest**

Steven Lehrer serves in a scientific advisory capacity with Fermata Pharma, Inc. Peter H. Rheinstein is President of Severn Health Solutions. The scientific content was developed without influence from Fermata Pharma Inc. or Severn Health Solutions.

